# Some Mechanisms Modulating the Root Growth of Various Wheat Species under Osmotic-Stress Conditions

**DOI:** 10.3390/plants9111545

**Published:** 2020-11-11

**Authors:** Nina V. Terletskaya, Tamara E. Lee, Nazira A. Altayeva, Nataliya O. Kudrina, Irina V. Blavachinskaya, Ulzhan Erezhetova

**Affiliations:** 1 Department of Biodiversity and Biological Resources, Faculty of Biology and Biotechnology, Al-Farabi Kazakh National University, Al-Farabi av., 71, Almaty 050040, Kazakhstan; irina-b-1952@mail.ru (I.V.B.); erezhetova@gmail.com (U.E.); 2 Institute of Plant Biology and Biotechnology, Timiryazev Str. 45, Almaty 050040, Kazakhstan; daizy-c@mail.ru; 3 Central Laboratory for Biocontrol, Certification and Preclinical Trials, Al-Farabi av., 93, Almaty 050040, Kazakhstan

**Keywords:** wheat species, primary roots, osmotic stress, root elongation, root anatomy, cytological reactions, superoxide dismutase

## Abstract

The role of the root in water supply and plant viability is especially important if plants are subjected to stress at the juvenile stage. This article describes the study of morphophysiological and cytological responses, as well as elements of the anatomical structure of primary roots of three wheat species, *Triticum monococcum* L., *Triticum dicoccum* Shuebl., and *Triticum aestivum* L., to osmotic stress. It was shown that the degree of plasticity of root morphology in water deficit affected the growth and development of aboveground organs. It was found that in conditions of osmotic stress, the anatomical root modulations were species-specific. In control conditions the increase in absolute values of root diameter was reduced with the increase in the ploidy of wheat species. Species-specific cytological responses to water deficit of apical meristem cells were also shown. The development of plasmolysis, interpreted as a symptom of reduced viability apical meristem cells, was revealed. A significant increase in enzymatic activity of superoxide dismutase under osmotic stress was found to be one of the mechanisms that could facilitate root elongation in adverse conditions. The tetraploid species *T. dicoccum* Shuebl. were confirmed as a source of traits of drought tolerant primary root system for crosses with wheat cultivars.

## 1. Introduction

Global climate change has led to irreversible phenomena that have significantly affected agriculture of many countries of the world [1,2], especially developing countries. Water scarcity has become a key and constantly increasing stressor in almost all climatic regions of the world. Understanding the mechanisms that regulate the response of plants to water scarcity is extremely important for improving the efficiency of agricultural crops and agroecosystems in the context of global climate change [3,4].

The role of root structure and architecture in water supply and plant viability cannot be underestimated and it is especially important if plants are subjected to stress at the juvenile stage [5]. Since roots grow underground, they are the first to perceive changes in external conditions such as water and nutrient content in soil, pH, temperature, etc., and adjust their genetic program for post-embryonic development to withstand the resulting stress [6]. These changes are integrated into the development program and cause various structural modulations and realignments of the root system architecture (RSA). With poorly understood mechanisms of genetic control of the processes, it is noted that the RSA of different species shows different levels of plasticity and reacts differently to adverse conditions [7]. The degree of interaction of the root with the environment is determined by different levels of response: molecular, cellular, histological, and organismal [8,9]. In the process of acclimation, the structure of the root system can be reconfigured by activating or declining the water potential, metabolism level, and enzymatic activity [10]. These changes can be both adaptively expedient and an expression of stress-related pathologies.

Cell growth, cell multiplication, and cell differentiation are inextricably linked, as already remarked by Torrey in 1956 [11], thus, only when the analysis of root growth is considered in terms of various components of cellular processes, is a detailed understanding of its mechanisms possible. However, there is still no consensus on the role of elemental growth processes, cell division, and expansion in the regulation both at root and plant level stress-responses [12]. Both the biophysical (hydraulic) and biochemical mechanisms of signaling systems that regulate linear root growth and structural modulations of RSA in conditions of insufficient water supply are still not fully defined [13]. It is possible that in-depth research of root system will contribute to a new “green revolution”, a significant increase in food security [14].

In this article, using the example of three different wheat species with different ploidy, we summarized some results obtained in the study of root architecture modulations and root elongation under water deficit. The aim of this article was to provide an analysis of how water deficit modulated post-embryonic primary roots of different wheat species development, how the individual morphological and anatomical elements of RSA of primary root were changed, and how the primary root apical meristem cells of different wheat species responded to osmotic stress. We also showed how the activity of important stress enzyme superoxide dismutase (SOD) was changed in primary roots of different wheat species under osmotic-stress conditions. 

## 2. Results

### 2.1. Morphophysiological Reactions of Primary Roots of Different Wheat Types to Osmotic Stress

The morphophysiological parameters per species varied significantly under stressful conditions. The least effect of water deficit was noted in *T. dicoccum*, significant in *T. aestivum*, and the greatest in *T. monococcum*.

**Figure 1:**
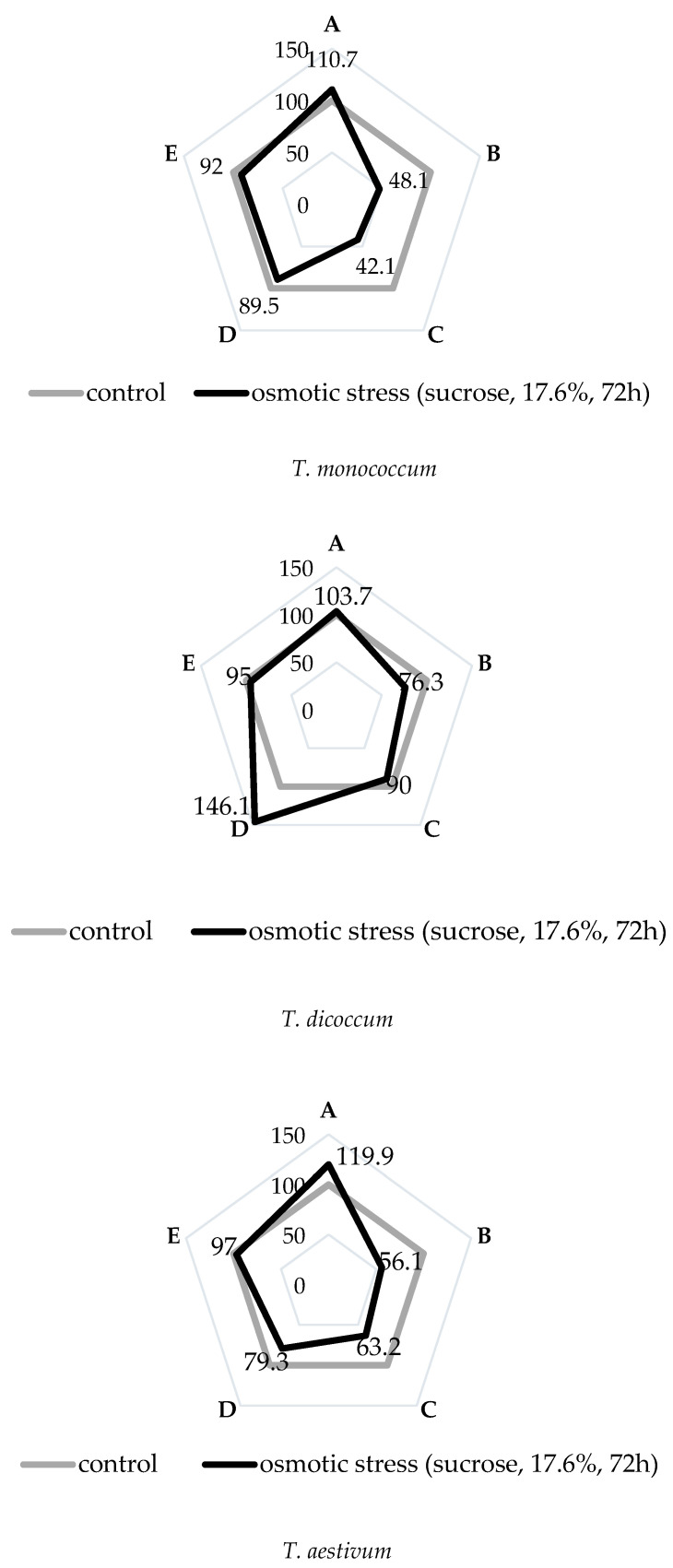
Morphophysiological parameters of primary roots of wheat species under induced water deficit. (A) root/shoot (length). (B) root relative water content. (C) root biomass. (D) root length. (E) number of roots.

From the data in Figure 1, it can be seen that the induced water deficit had a negative impact on the morphophysiological parameters of the studied wheat species, but in *T. dicoccum* species the damaging effect of osmotic stress on the studied parameters was minimal. Although its value of relative water content (RWC) decreased slightly, the root length in stressful conditions significantly increased in comparison with the control plants. The species *T. monococcum* was characterized by maximum decrease in RWC and biomass indicators during water deficit. We also noted the rise of root/shoot length ratio in *T. monococcum* and *T. aestivum* species under stress. At the same time we noted the absence of significant differences in the number of roots under stress and control conditions in all the studied plant species.

### 2.2. Changes in the Elements of the Anatomical Structure of Different Wheat Species’ Primary Roots during Osmotic Stress 

Upon cross-section, anatomical structures such as the epiblem, primary cortex, and central cylinder were clearly visible (Supplementary Figure S1A). The cell layers’ number was invariable in all species along the entire root length. Staining by Sudan III made it possible to identify areas of probable suberization, which were stained orange (Supplementary Figure S1B,C).

As follows from the data presented in Table 1, the increase in root diameter along with the increase in ploidy of the wheat species were noted under control conditions. Nevertheless, under conditions of induced water deficit, the thickness of the roots of all studied species increased in equal proportions, totaling 110% of the control for each species. Meanwhile, the increase in the root diameter in different species was due to different components.

In *T. monococcum*, this was facilitated by the thickening of the exoderm. In *T. dicoccum,* a thickening of the exoderm also occurred, but thickness of the epiblem was significantly reduced, as well as the thickening of cortical parenchyma and endoderm. Moreover, in *T. aestivum*, the root diameter was enlarged under stress due to the significant thickening of the endoderm and the increase in the diameter of central cylinder.

**Figure 2:**
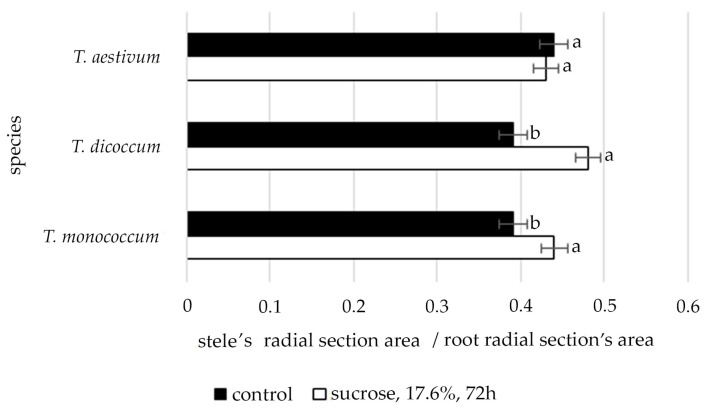
The changes of the stele’s radial section area/root radial section’s area ratio of wheat species under induced water deficit (sucrose, 17.6%, 72 h). Values presented are means (±SD). Different letters above the bars represent significant differences at *p* ≤ 0.05, *n* = 5 plants in each of 3 replicates for all treatments.

The data shown in Figure 2 clearly demonstrate that only in species *T. aestivum,* under conditions of induced water deficit, the ratio of radial cross-section area of the stele to the radial cross-section area of the root was stable. In the other two studied species, this ratio was increased significantly.

### 2.3. Cytological Reactions of Primary Roots of Different Types of Wheat to Osmotic Stress

**Figure 3:**
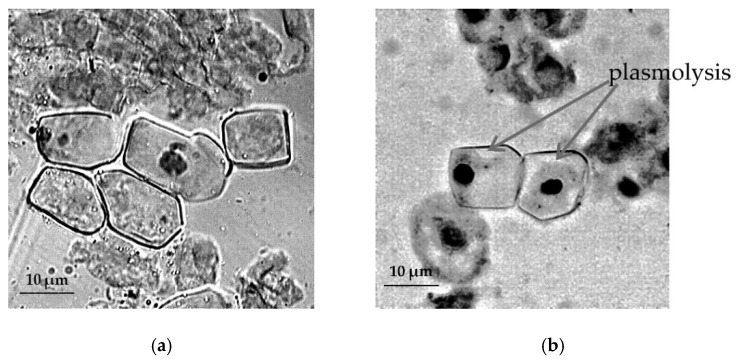
The effect of induced water deficit on root apical meristem (RAM) cells *T. monococcum*: (**a**), control, (**b**), stress (sucrose, 17.6%, 72 h), scale bar = 10 µm.

The study of cytological reactions of primary roots shows pronounced plasmolysis of root apical meristem (RAM) cells of *T. monococcum* species under stressful conditions (Figure 3).

**Figure 4:**
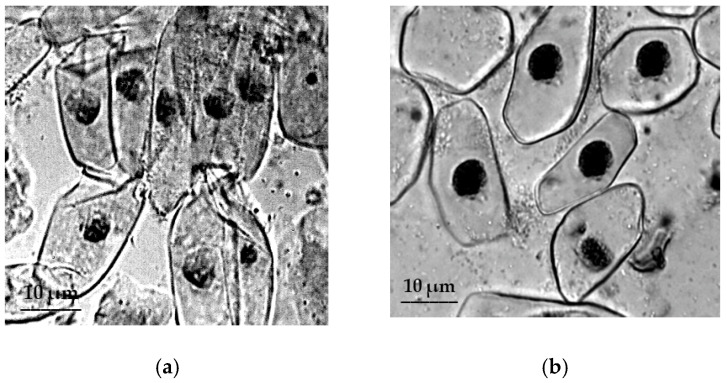
The effect of induced water deficit on RAM cells *T. dicoccum*: (**a**), control, (**b**), stress (sucrose, 17.6%, 72 h), scale bar = 10 µm.

The meristem cells of the primary roots of *T. dicoccum* retaining turgor under the conditions of osmotic stress (Figure 4). 

**Figure 5:**
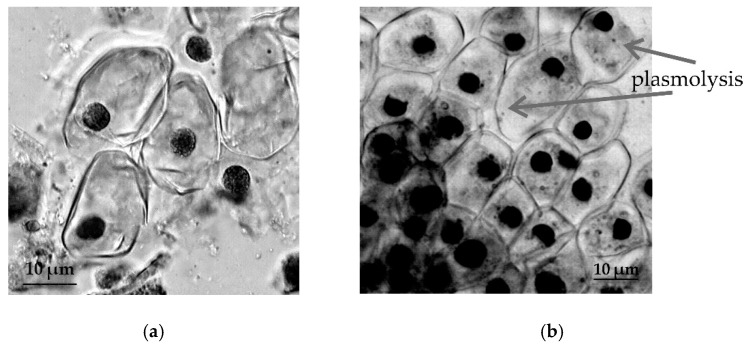
The effect of induced water deficit on RAM cells *T. aestivum*: (**a**), control, (**b**), stress (sucrose, 17.6%, 72 h), scale bar = 10 µm.

**Figure 6:**
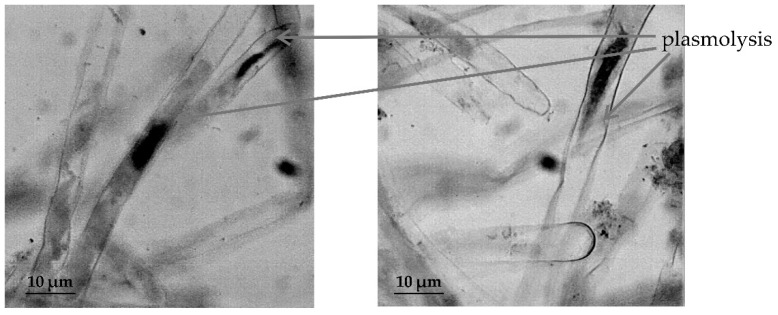
The effect of induced water deficit on root hairs cells *T. aestivum* (sucrose, 17.6%, 72 h), scale bar = 10 µm.

In *T. aestivum* both in RAM cells (Figure 5) and in the cells of root hairs (Figure 6), the development of the process of plasmolysis was noted. 

### 2.4. Changes in Superoxide Dismutase Activity in Primary Roots of Different Wheat Species during Osmotic Stress

**Figure 7:**
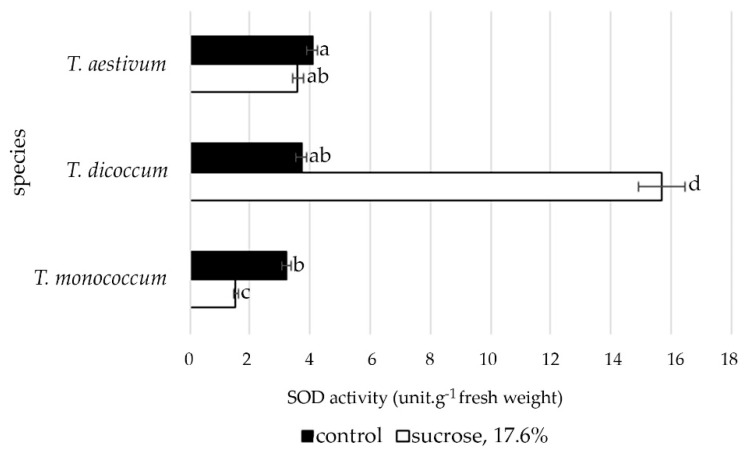
The SOD activity in primary roots of wheat species under induced water deficit (sucrose, 17.6%, 72 h). Values presented are means (±SD). Different letters above the bars represent significant differences at *p* ≤ 0.05, *n* = 25 plants in each of 3 replicates for all treatments.

The data presented in Figure 7 show that the activity of superoxide dismutase (SOD) in the roots of *T. monococcum* and *T. aestivum* differed significantly, but there was a slight difference from *T. dicoccum* under control conditions. However, induced water deficit revealed significant differences in the activity of this antioxidant enzyme. In *T. monococcum* and T*. aestivum* species, there were reductions in SOD activity to varying degrees: in *T. monococcum* the decrease was significant, and in *T. dicoccum* the level of antioxidant activity of SOD increased significantly.

## 3. Discussion

Maintaining root growth in conditions of water scarcity at the early stages of ontogenesis is important for plant survival, since longer roots in drying soil have additional advantage—they make water and mobile nutrients more accessible [15,16]. Quite often, in stressful conditions, monitoring water uptake by the root is more important for overcoming the traumatic effects of osmotic stress than regulating leaf transpiration [13]. However, in long-term breeding practice, the selection of plants is based mainly on indicators of bio-productivity of organs, which has led to the decrease in biomass of roots of modern varieties compared to drought-resistant wild species [17]. This is confirmed by the results obtained: the studied species were ranked as follows: *T. aestivum* < *T. monococcum* < *T. dicoccum* by the value of root length index in the conditions of induced water deficit compared to the control.

The degree of plasticity of the root morphology affects the growth and development of aboveground organs [18,19]. This was evidenced, in particular, by the data on changes in the root/shoot ratio under stressful conditions, shown in Figure 1, on *T. dicoccum*; the value of this indicator remained almost at the level of the control value during water deficit. Whereas the rise of root/shoot index in *T. monococcum* and *T. aestivum* species in conditions of induced water deficit indicated the negative effect of this stress on the growth parameters of the first leaves of these species. Ranking by this attribute (% to control) is as follows: *T. dicoccum* < T*. monococcum* < *T. aestivum*.

What mechanisms can cause changes in root response to osmotic stress?

Although the influence of osmotic stress on the development of root system has been studied for a long time, very few studies are devoted to mechanisms that affect the change in the rate of root growth during water deficit. Osmotic stress certainly leads to dehydration of plant tissues. Yang et al. [20] showed that the growth of root cells depended primarily on their osmotic potential and turgor. A slight loss of water in the root could increase tensile strength, while a significant loss of water can lead to a decrease in the root’s ability to elongate.

All studied species showed a tendency towards the decrease in the RWC of the root under conditions of induced water deficit relatively to the control. They can be arranged as follows: *T. monococcum* < *T. aestivum* < *T. dicoccum*. The tetraploid species *T. dicoccum* was characterized by maximum water content of the primary roots in relation to the control compared with the other studied species. Therefore, the root’s ability to retain water under osmotic stress is an important factor determining its growth.

A similar ranking of studied species was obtained while analyzing the accumulation of biomass by primary roots in water deficit conditions relative to the control. It is logical to say that osmotolerant species have larger, stronger, and deeper roots in contrast to sensitive ones [21].

The literature shows that roots use various morphophysiological developmental strategies. For example, they can change the growth rate, diameter, and density of tissue, adapting to various stressors [22,23,24]. At the same time, the range of changes in the root diameter within the root system of a specific species can also vary [25]. However, a too small root diameter restricts root penetration through soil and does not contribute to the development of internal structures that transport water and nutrients [26,27]. However, there is evidence that these signs also affect the ability of the root to stretch [20]. In particular, as shown by Genet et al. [28], negative correlations of the root diameter with tensile strength and positive correlations with its tensile resistance were found. Qian Wu et al. [29] state that the basal diameter of the root determines its potential length. As a rule, the longest roots are those that maintain (and sometimes increase) their diameters during elongation.

In our experiment, no such correlation was found. It was noted that a rise in the absolute values of root diameter increased with the rise in the ploidy of wheat species. However, relative values under induced water deficit were the same for all the studied species and amounted to 110% compared to the control.

Changes in anatomical characteristics in stress conditions suggest maintaining some balance that is supposed to be between the diameter and adaptive capacity of roots. According to the literature and our own research [9,30], different abiotic stresses have a range of effects on RSA. In the case that salt stress caused a significant reduction in root diameter of various wheat species [30], water deficit triggered a significant enlargement in the diameter of primary root of all the studied species. At the same time, we witnessed a significant increase in the thickness of exoderm in the osmotic stress conditions in species *T. monococcum* and *T. dicoccum*. A thickening of the endoderm was observed in the species *T. dicoccum* and *T. aestivum*. This can be explained largely by the ongoing suberization of roots, i.e., the formation of suberized barriers in certain layers of cell walls, such as endo- and exoderm [31]. As shown by numerous literature data, the vital activity of the root under stressful conditions largely depends on such heteropolymers as suberins, which are deposited in cell walls to separate living plant tissue from the negative impact of environment and optimize the function of resource absorption [24,32,33,34]. For example, Baxter et al. [35] found in *Arabidopsis* Esb1 mutants that were characterized by increased root suberization, reduced daily transpiration rate, and increased water use efficiency during the vegetative growth period. The decrease in thickness of epiblem in *T. dicoccum* may have been due to mechanisms of compensating this cell layer by exoderm.

One of the indicators of the adaptation process is the ratio of thickness of exoderm to endoderm [36,37]. Taking into account the data in Table 1, in our experiment the value of this ratio in induced water deficit conditions rose in the drought-sensitive species *T. monococcum* (0.8–1.5), practically did not change in drought-tolerant *T. dicoccum* (1.3–1.2) and declined in *T. aestivum* (1.5–1). Moreover, this allows us to recognize different types of strategies for stress response of the root.

Cortical parenchyma cells are highly vacuolated. This creates turgor pressure and allows the root to retain the necessary amount of water and nutrients. On the one hand, increasing the thickness of cortical parenchyma under stress implies the longer radial distance for transporting water at the root. On the other hand, the thickening of this layer may indicate the adaptive strategy for water conservation, as we observed in the drought-tolerant *T. dicoccum*.

Tolerance to osmotic stress can also be provided by a thicker root stele, and, respectively, by thicker xylem vessels and greater water conductivity. Stele diameter enlargement, and consequently, the cross-sectional area of stele during water stress contributes to decline in radial path for water flow and higher axial conductivity [38,39]. A clear illustration of this is the ratio of area of radial section of stele to the area of radial section of root [39]. In the present study, this pathway of root adjustment to water deficit was only observed in *T. aestivum*. However, an incidence of runaway embolism might also increase in thicker vessels in response to drought, which will lead to a higher rate of plant damage and mortality [40]. Thus, anatomical modulations of the root, including possible suberization, which under stressful conditions play an important role in water absorption and contribute to prevention of water loss (backflow) from root to source of drought [41], are species-specific.

The growth rate of primary root largely determines the growth of aboveground organs and plant productivity [42,43]. The viability of the RAM, which provides differences in the size and mass of the plant, and the very possibility of its normal functioning in a changing environment is of particular importance in stressful conditions [44]. Root elongation is the result of both cell enlargement and mitotic activity of apical meristem cells, which are constantly formed due to their division. Attempting to link root elongation to cellular processes, Shimazaki et al. [45] concluded that an insufficient water supply in apical meristem zone could lead to significant reduction in root growth. Stress affects the organization of root apical meristem [46]. It is shown that under strong osmotic stress, programmed death from autophagy is possible in the cells of RAM [47]. Duan et al. [48] suggested that under conditions of insufficient water supply, plants could activate root apical meristem cell death program, thus eliminating apical dominance and rearranging RSA to better adapt to a stressful environment.

Our study in 2019 revealed significant species-specific differences in the cytological responses of RAM cells of various wheat species to salt stress. Moreover, the negative effect of salt stress was observed not only on water content in root cells, but also on their chromosomal apparatus of sensitive wheat forms [30]. The results of induced water deficit did not show such a destructive effect on the studied wheat species, but in meristematic cells of primary roots of *T. monococcum* and *T. aestivum* we observed the development of strong plasmolysis under osmotic stress conditions. In *T. aestivum*, strong plasmolysis was also observed in root hair cells, whose purpose in RSA is to significantly enlarge the root surface area, increasing absorption of water and soil solutions into the root [49,50]. Thus, the result of cytological observations may indicate an early symptom of viability loss due to water deficit in *T. monococcum* and *T. aestivum*. In drought-tolerant species *T. dicoccum*, RAM cells showed sufficient turgor under conditions of induced water deficit.

There are references in the literature that various abiotic stressors can induce the intense production of reactive oxygen species (ROS) in plants. To some extent, ROS always presents in plant tissues, since it is necessary for regulating signaling and growth [51]. Various stressors show that abiotic factors induce the presence of ROS in plant tissues, which leads to change in root morphology, first of all due to reduced growth of the primary root, but mechanisms of such redox generation are still not well understood [52,53,54]. Nevertheless, it is the degree of ROS accumulation that can be the indicator of adaptation or damage degree in plant tissues [55,56].

ROS fluctuations in time and space are often interpreted as stress signals to regulate cell growth, development, or death [57,58]. Miller et al. [53] suggested that the production of ROS is a necessary element of reaction for the adequate acclimatization of plants to stress processes. This effect results from the expression of several genes encoding antioxidant enzymes, such as superoxide dismutase (SOD), which can convert superoxide radicals into hydrogen peroxide, water, and oxygen.

Osmotic stress can affect accumulation of ROS in plant roots because it results in lipid peroxidation in the plasma membrane. The research by Libik-Konieczny et al. [59] on rhizogenesis showed the importance of redox balance system for this process, which could be mediated by preventing the negative effects of possible oxidative stress. Isoforms of superoxide dismutase (SOD) and peroxidase (POD) can play a significant role in regulating the content of hydrogen peroxide in the formation of root primordia, as well as in the process of root growth and development [59,60]. The accumulation of ROS in meristematic cells of root tips subjected to stress serves as a signal for autophagy [61]. ROS quenching inhibits root growth [62] and an increased peroxidase expression contributes to root elongation [63]. Considering experimental data of the present study, we can state that the increased activity of SOD in primary roots of *T. dicoccum* under induced water deficit conditions indicates the greater tolerance of this species to osmotic stress at the early stages of ontogenesis and is one of the mechanisms that can provide root elongation in unfavorable conditions.

## 4. Materials and Methods

Three winter wheat species with different ploidy were used in the study: *Triticum monococcum* L. (A^u^B), *Triticum dicoccum* Schuebl. var. araratum (Host) Koern (A^u^A^u^BB), and *Triticum aestivum* L. (A^u^A^u^BBDD)—Mironovskaya 808. All originated from the Institute of Plant Biology and Biotechnology collection (Almaty, Kazakhstan). The tetraploid species *T. dicoccum* was previously characterized one having a number of physiological parameters of salt and drought tolerance in in addition to being a carrier of Dreb-B1 drought tolerance gene [30,64].

### 4.1. Analysis of Physiological Parameters of Drought Tolerance

During the seedling stage, all wheat species were subjected to simulated drought. First, the studied wheat species and lines were germinated in a growth chamber in the dark at 25 °C for 3 days. Then, some of seedlings (25 plants in each from 3 biological replicates) were grown in vessels with distilled water under circadian illumination (using commercial fluorescent white light tubes): 16 h light/8 h darkness regime [200 µmol m^−2^ s^−1^ PAR, light metre LI-205 (Li-Cor, Lincoln, NE, USA)], and 26 ± 2 °C temperature. Some of the seedlings (25 plants in each from 3 biological replicates) were transferred to 17.6% sucrose solution (*w*/*v*) with osmotic potential (ψ) = 1450 kPa for 72 h. The roots were not exposed to light; they were protected from light by a plastic screen with holes for shoots [65].

The elongation of primary root, first leaf (сm), and their ratio (%) was measured after 72 h both in control and stress-treated replicates. The wet and dry biomass of primary roots was measured.

The relative water content (RWC) of the roots was calculated by the formula:

RWC = ((а − b):а) × 100%, where a—the initial wet mass of roots (mg); b—dry mass (mg) of roots after drying; samples (a roots clipped) was dried in a forced air oven at 105 °C, 5 h [65].

All experiments were done in three replicates. The processing of data and graphing was performed using Microsoft Excel (Microsoft Corp., Redmond, Washington, DC, USA). Atypical values were excluded from the data based on *t*-tests, the standard error of the average sample was calculated. Differences were considered significant at *p* < 0.05.

### 4.2. Anatomical Analysis of Primary Roots

The fixation of roots was performed in 70% ethanol, рreservative fluid was a Strasburger-Flemming’s mixture: 96% ethanol: glycerol: water in ratio of 1:1:1 [66]. Anatomical specimens were prepared with a microtome MZP-01 (“Technom”, Ekaterinburg, Russia) having a freezing unit OL-ZSO 30 (“Inmedprom”, Yaroslavl, Russia). The thickness of anatomical sections varied between 10 to 15 microns. Sudan III –stained sections were placed on a glass slide in a drop of pure glycerin and covered with a cover slip to obtain a temporary preparation. Micrographs of anatomic sections were made on a microscope with MC 300 (Wien, Austria) CAM V400/1.3M camera “Vision” (Wien, Austria). All anatomical data were obtained in 3–5 replicates (5 plants in each) with a 40× objective.

### 4.3. Cytological Analysis of Primary Roots

Cytological analysis was carried out with a squashed preparation (root tips 1–1.5 cm long from the primary roots) stained with Acetocarmine by the method of Pausheva [67]. The material was fixed in the morning hours in freshly prepared Clark’s reagent (3 parts of 96% ethanol: 1 part of glacial acetic acid) for 12–24 h. After fixation and storage in a refrigerator, the primary roots were warmed at room temperature for several hours, transferred into a dye, and heated in a boiling water bath for 6 min. The roots were left in a test tube with acetocarmine at room temperature for 30 min for better staining of the meristem. The root tips were placed on a defatted (ethanol-treated) glass slide in a drop of 45% acetic acid and squashed for short-term slides. All cytological examination was conducted by a Micros microscope (Graz, Austria), photographed with a YONGXIN OPTICS CAM V200 (Ningbo, China) video camera and YONGXIN OPTICS ScopePhoto version 2.4 computer program with an increase in lens × 40. All data were obtained in three replicates. All slides were analyzed in at least five fields of view.

### 4.4. Analysis of Superoxide Dismutase (SOD) Activity

Fresh whole roots 0.5 g were homogenized with a mortar and pestle in 4.5 mL ice*-*cold 30 mm K/Na *-*phosphate buffer pH 7.4 containing 0.1 mm EDTA and 2% polyvinylpyrrolidone (PVP mol. mass of 25,000) for 3 min. After filtration through kapron the homogenate was centrifuged at 11,000× *g* for 20 min, and the supernatant diluted 20 times by K/Na *-*phosphate buffer (without EDTA and PVP) was used as the source of enzymes. All the steps were carried out at 0–4 °C.

The activity of SOD was determined by photoreduction of nitroblue tetrazolium chloride (NBT) dye (Sigma-Aldrich, St. Louis, MO, USA) in the presence of riboflavin and methionine generating superoxide anion radicals (O^2−^). The blue formazane produced by NBT photoreduction was measured as the increase in absorbance at 560 nm. The reaction mixture (3 mL) contained 1.3 μM riboflavin, 63 μM methionine in 50 mM K/Na phosphate buffer with 0.1 mM EDTA, pH 7.4 and 0.1 mL enzymatic extract (all reagents Sigma-Aldrich, MO, USA). The reaction took place in a chamber under illumination of a 30 W fluorescent lamp at 25 °C. The reaction was initiated by turning the fluorescent lamp on and stopped 6 min later by turning it off [68]. The measurements were made on a spectrophotometer LEKI SS2107UV (MEDIORA OY, Helsinki, Finland). As a unit of SOD activity, the volume of enzymatic extract was taken, which caused 50% inhibition of photoconverted NBT. One SOD unit was defined as the amount of enzyme required to inhibit 50% of the NBT photoreduction in comparison with tubes lacking the plant extract [69]. The determination of SOD activity (in relative units per milligram of protein) was carried out three times using the following formula:

SOD activity (unit·g^−1^ fresh weight) = lg(Dc/Do)/(lg2 × Сp), where Dc—optical density of the light control sample; Do—optical density of the experimental sample; Cp—protein content in the sample, mg/mL.

The soluble protein content in the supernatant was determined by Lowry [70].

All data were obtained in three replicates (25 plants in each replicates). The significance of differences based on *t*-tests, the standard error of the average sample, were calculated. Differences were considered significant at *p* < 0.05.

## 5. Conclusions

The results of this study demonstrated that under osmotic stress conditions, morphophysiological and cytological responses as well as elements of anatomical structure of primary roots changed at various degrees in different wheat species, which reflected species-specific features and could be used to assess their ecological adaptability.

Significant increase in enzymatic activity of SOD under osmotic stress can be considered as one of the mechanisms that can facilitate root elongation in adverse conditions. 

The tetraploid species *T. dicoccum* Shuebl is recommended as a source of such traits of drought-tolerant root system as ability to optimize anatomical structures, preservation of the turgor of RAM cells, and high SOD activity under water stress at the juvenile stage of development, for crosses with wheat cultivars. 

## Figures and Tables

**Table 1:** Influence of water deficit on the anatomical parameters of the roots of different wheat species.

Condi-tions	Parameters					
	Thickness of the Epiblem, μm	Thickness of the Exoderm, μm	Thickness of the Cortical Parenchyma, μm	Thickness of the Endoderm, μm	Diameter of the Central Cylinder (Stele), μm	Diameter of the Root, μm
*T. monococcum*						
control	14.8 ± 0.7	13.9 ± 0.4	74.9 ± 1.7	18.6 ± 0.6	191.4 ± 2.2	435.8 ± 7.1
sucrose, 17.6%	14.1 ± 0.7	27.7 ± 0.2 *	85.1 ± 1.9 *	18.6 ± 0.5	187.6 ± 1.4	478.5 ± 2.2 *
% to control	95	199	113	100	98	110
*T. dicoccum*						
control	19.4 ± 0.2	16.7 ± 0.8	67.3 ± 0.4	12.8 ± 0.6	217.5 ± 3.5	449.9 ± 5.2
sucrose, 17.6%	13.7 ± 0.1 *	30.4 ± 0.6 *	80.0 ± 0.7 *	26.4 ± 0.8 *	192.9 ± 1.1 *	493.9 ± 0.6 *
% to control	71	182	119	206	89	110
*T. aestivum*						
control	16.6 ± 0.8	28.5 ± 0.9	70.1 ± 1.2	19.7 ± 0.5	205.8 ± 3.6	475.5 ± 9.2
sucrose, 17.6%	18.5 ± 0.1 *	25.6 ± 0.8 *	75.9 ± 0.5 *	24.5 ± 0.3 *	234.0 ± 8.9 *	523.0 ± 9.8 *
% to control	112	90	108	124	114	110

Note: * indicate significant differences at *p* ≤ 0.05, *n* = 5 plants in each of 3 replicates for all treatments.

## Supplementary Materials

The following are available online at https://www.mdpi.com/2223-7747/9/11/1545/s1, Figure S1: Anatomical structure of wheat roots under induced water deficit (sucrose, 17.6%, 72h). (A) Epiblem. (B) Exoderm. (C) Cortical parenchyma. (D) Endoderm. (E) Central cylinder (stele). (S) Suberization. Scale bar = 20 µm.
