# Supplementary material for: Some Mechanisms Modulating the Root Growth of Various Wheat Species under Osmotic-Stress Conditions
Source: Plants (Basel). 2020 Nov 11;9(11):1545. doi: 10.3390/plants9111545 (PMC7696822; doi:10.3390/plants9111545)
Supplement: Supplementary file 1 [file plants-09-01545-s001.pdf]

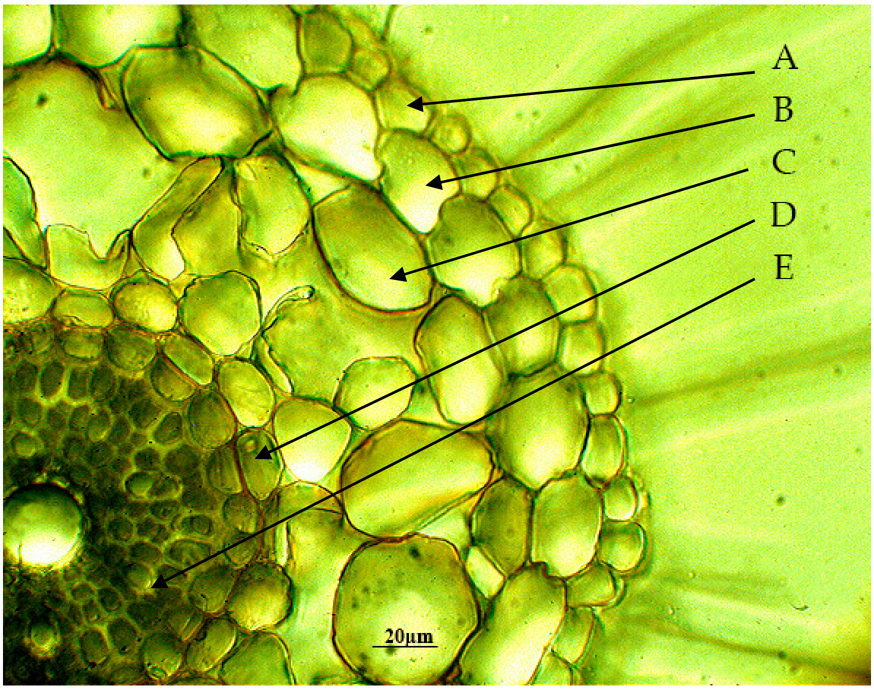


*T. monococcum*, control

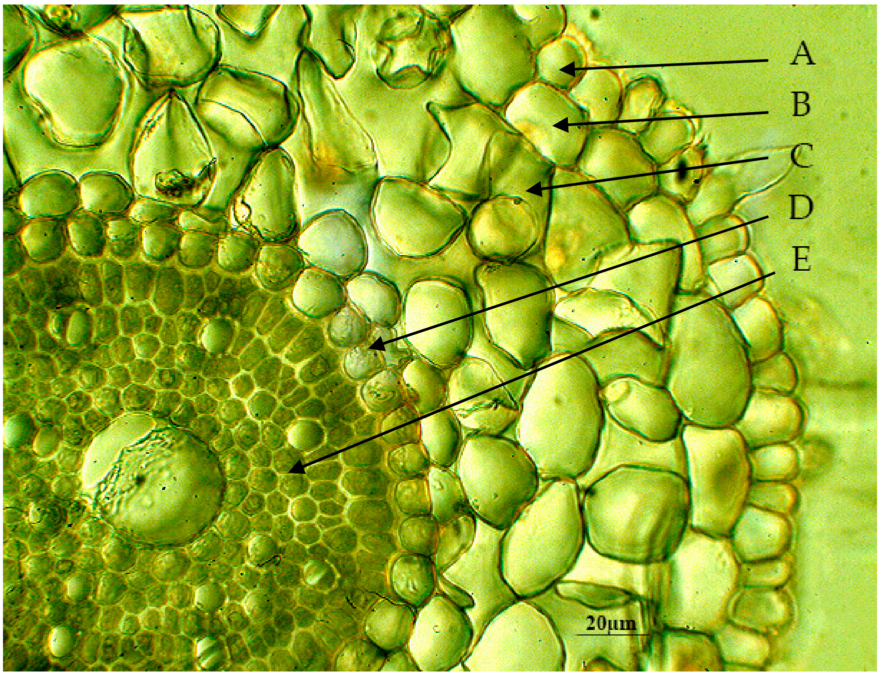


*T. monococcum*, (sucroce, 17.6%, 72 h)

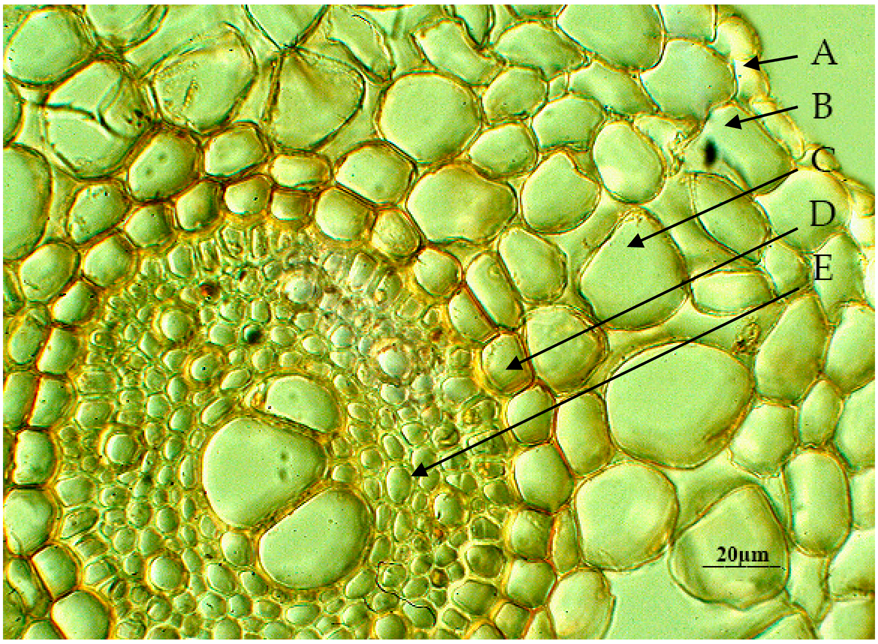


*T. dicoccum* control

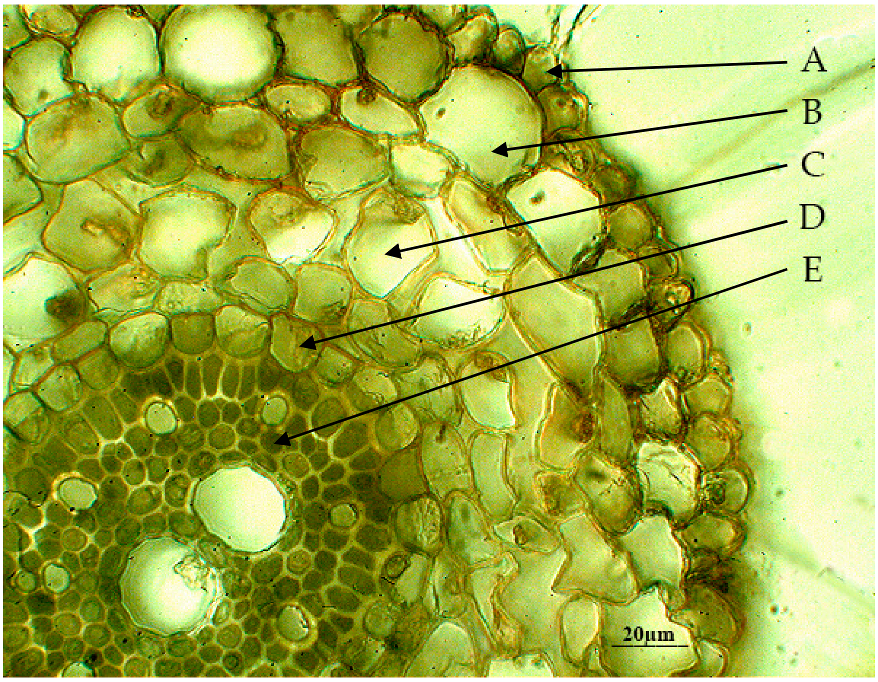


*T. dicoccum*, water stress (sucroce, 17.6%, 72 h)

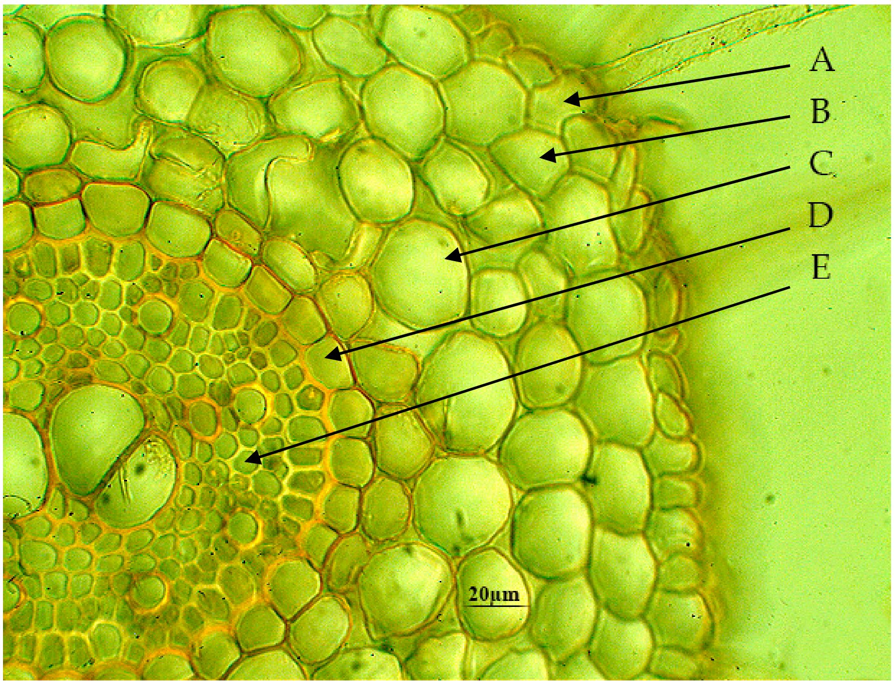


*T. aestivum*, control

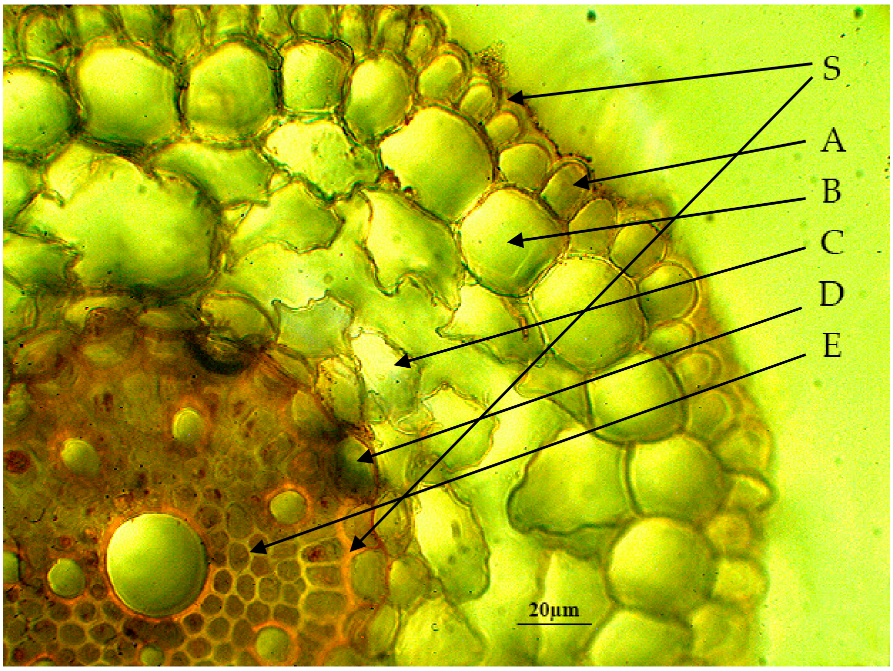


*T. aestivum*, water stress (sucroce, 17.6%, 72 h)

**Supplementary Figure S1.** Anatomical structure of wheat roots under induced water deficit (sucrose, 17.6%, 72h). **(A)** Epiblem. **(B)** Exoderm. **(C)** Cortical parenchyma. **(D)** Endoderm. (**E**) Central cylinder (stele). (**S**) Suberization. Scale bar = 20 µm
